# *Zanthoxylum* alkylamides ameliorate protein metabolism disorder in STZ-induced diabetic rats

**DOI:** 10.1530/JME-16-0218

**Published:** 2017-01-18

**Authors:** Tingyuan Ren, Yuping Zhu, Xuejuan Xia, Yongbo Ding, Jing Guo, Jianquan Kan

**Affiliations:** 1 College of Food ScienceSouthwest University, Chongqing, China; 2 Laboratory of Quality & Safety Risk Assessment for Agro-products on Storage and Preservation (Chongqing)Ministry of Agriculture, Chongqing, China; 3 Institute of Biological EngineeringChongqing University, Chongqing, China; 4 Department of NutritionDaping Hospital & Research Institute of Surgery, Third Military Medical University, Chongqing, China

**Keywords:** *Zanthoxylum* alkylamides, diabetes, protein synthesis, catabolism

## Abstract

This study aimed to evaluate the protein metabolism effect of *Zanthoxylum* alkylamides and to explore the potential mechanism in streptozotocin (STZ)-induced diabetic rats. Diabetic rats were orally treated with 2, 4 and 8 mg per kg bw of alkylamides daily for 28 days. Alkylamides decreased the relative weight of the liver and food intake, significantly increased the relative skeletal muscle weight and significantly decreased the blood urea nitrogen levels. Insulin, insulin-like growth factor 1, total protein (TP) and albumin (ALB), globular proteins and ALB proteins/globulin protein levels in serum significantly increased. TP, RNA content and RNA/DNA ratio significantly increased in the skeletal muscle of diabetic rats. Real-time quantitative polymerase chain reaction results indicated that alkylamides significantly increased the mRNA expression of insulin receptor (InR), IGF1 and insulin-like growth factor 1 receptor (IGF1R) in the liver and skeletal muscle. Moreover, the mRNA and protein expression levels of PI3K, PKB and mTOR significantly increased, whereas those of atrogin-1, muscle ring finger 1 and FOXO in the skeletal muscle significantly decreased. Alkylamides may advance protein synthesis by the PI3K/PKB/mTOR signalling pathway and attenuate the catabolism of protein through the ubiquitin–proteasome pathway. Therefore, it was possible that alkylamides ameliorate protein metabolism disorders in diabetic rats by activating the mTOR pathway.

## Introduction

As a global disease, diabetes mellitus seriously endangers human health. It is a glucose, fat and protein metabolism disorder caused by the absolute or relative deficiency in insulin secretion (Michaelides *et al*. 2016). According to the latest statistics of the International Diabetes Federation, an estimated 415 million adults have diabetes, and 1 of every 11 adults in the world is diagnosed with this disorder. By 2035, the number of diabetics is expected to increase to 592 million, approximately 10% of the total population (IDF, http://www.diabetesatlas.org/). Currently, injection of insulin and oral hypoglycaemic drugs, including sulfonylureas, glinides, biguanides, α-glucosidase inhibitor and dipeptidyl peptidase-IV inhibitor (Inzucchi 2002, Zhu *et al*. 2013), is considered the primary treatment for diabetes. However, several problems have emerged, such as drug side effects, drug resistance and drug safety. Therefore, the research and development of natural substances for the safe and efficient treatment of diabetes is important.

Pepper (*Zanthoxylum bungeanum* Maxim) is a Rutaceae *Zanthoxylum* (Rutaceae) plant fruit mainly distributed in the Sichuan, Chongqing, Hunan, Shaanxi and Shandong Provinces of China and some Southeast Asian countries. Pepper is commonly used in traditional Chinese medicine and is among the eight main condiments in China. Current research shows that *Zanthoxylum* alkylamides mainly comprise α-, β- and γ alkylamides, which have antioxidant (Batool *et al*. 2010, Xia *et al*. 2011), anticancer (Chou *et al*. 2011) and hepatoprotective (Guo *et al*. 2011) effects.

Previous research showed that for rats with a lipid metabolism disorder induced by a high-fat diet, the expression levels of sterol regulatory element-binding protein 2 and 3-hydroxy-3-methylglutaryl-coenzyme reductase in the liver and the ileal bile acid-binding protein and sodium-dependent bile acid transporter in the ileum were significantly reduced by alkylamides. By contrast, the expression levels of transient receptor potential vanilloid subtype 1 (TRPV1) in the liver and ileum were significantly increased by the same kind of reagents. These results revealed that lipid metabolism disorder induced by a high-fat diet was improved by alkylamides (Yu 2015). In addition, fasting blood glucose and plasma fructose levels significantly decreased in diabetic rats, and oral glucose tolerance was improved. Total cholesterol (TC) and triglyceride (TG) levels in the plasma and fat, as well as TC and TG concentrations in the liver, were effectively reduced by alkylamides. These alkylamides prevented the accumulation of fat droplets in the liver of diabetic rats. Alkylamides can also significantly increase the expression of pancreatic–duodenal homeobox 1 (PDX-1), glucose transporter-2 (GLUT2), glucokinase (GK) and TRPV1 in the pancreas of diabetic rats and downregulate the expression of cannabinoid receptor l (CB1) in the liver and pancreas (Dossou *et al*. 2013, You *et al*. 2015). This explanation indicates that alkylamides can inhibit gluconeogenesis, reduce glycogen output, repair islet function and promote insulin secretion (Oh *et al*. 2013). However, diabetes is not only characterised by elevated blood glucose and blood lipid levels, but it also causes a protein metabolic disorder (Michaelides *et al*. 2016). Alkylamides can promote insulin secretion (You *et al*. 2015), which can promote protein synthesis by activating the PI3K/Akt/mTOR signalling pathway and inhibiting protein decomposition (Lim *et al*. 2016). However, the effect of alkylamides on protein synthesis and decomposition in diabetic rats has not been reported. Protein synthesis and decomposition have been found to be dependent on the mammalian target of rapamycin (mTOR) pathway. This observation implies that mTOR can be used as a cross-site of protein synthesis and decomposition process. mTOR plays a role in regulating protein metabolism by controlling upstream and downstream molecules (Fogg *et al*. 2011). The ubiquitin–proteasome pathway (UPP) connects the ubiquitin to the target protein by a three-enzyme cascade. UPP is the most vital and selective protein degradation pathway in mammalian cells, and it mediates the degradation of 80–85% protein in eukaryotes (Chen *et al*. 2012, Lu *et al*. 2013). Therefore, streptozotocin (STZ)-induced diabetic rats were used as models to explore the effect of alkylamides on protein metabolism disorder and its mechanism of action. These animal models provided reference for the comprehensive evaluation of the role of alkylamides in diabetic treatment.

## Materials and methods

### Alkylamide extraction and identification

Following the method of You and coworkers (You *et al*. 2015), 20 g of *Z. bungeanum* oleoresin and 40 g of thin-layer chromatography silica gel (GF-254) activated for 1 h at 110°C were stirred and mixed together. A total volume of 200 mL of absolute ethyl ether was added to the mixture. The mixture was then sealed and mixed with a magnetic mixer for 2 h prior to filtration. The filtrate was discarded afterward. The upper layer was dried and methanol five times its volume was added. The mixture was then sealed and extracted for 6 h in a water bath at 55°C prior to filtration. The filtrate was discarded and the extract was collected. The extract was steam-dried by a rotary evaporator. Up to 50 mL of ether was added at a boiling range of 30–60°C. Then, reflux extraction was performed for 10 h in a water bath at 50°C. The supernatant was frozen and crystallised in a −20°C refrigerator.

Purity analysis of alkylamides was performed by a high-performance liquid phase (Agilent 1260). Exactly 0.05 mg alkylamides dried by nitrogen was dissolved in methanol at a volume of 5 mL and filtered through a 0.45 µm microporous membrane. The samples were applied to a C18 column (4.6 mm × 250 mm, 5 µm, Agilent). Mobile phase A had 50% water and mobile phase B had 50% methanol. The flow rate was 1 mL/min, sample volume was 10 µL, column temperature was 40°C and ultraviolet detection wavelength was 254 nm.

### Diabetes induction and experimental design

This study was conducted in strict compliance with the recommendations of the State Scientific and Technological Commission and with the Guide for Sichuan Laboratory Animal Management Committee (in Chinese). The protocol was approved by Chengdu Qiankun Animal Pharmaceutical Co. Ltd. (Permit Number, SYX (Chua) 2014-190). Eight-week-old male Sprague–Dawley rats were acquired from Chongqing Tengxin Laboratory Animals, Inc. (Chongqing, China; animal licence SCXK 2012-0008). The rats were housed individually in stainless steel metabolism cages (25 cm × 15 cm × 15 cm) in a temperature- and humidity-controlled room maintained at 25 ± 2°C with 12 h/12 h light/darkness cycles. The rats were allowed free access to a standard diet (Chongqing Tengxin Laboratory Animal, Inc., Chongqing, China) and water *ad libitum*. The rats were acclimatised to the laboratory environment for one week.

After subjected to overnight fasting, the rats were intraperitoneally injected with 60 mg per kg bw of STZ (Sigma Chemicals) dissolved in 0.1 mol/L of citrate buffer (pH 4.5) (Qiao *et al*. 2014). The rats in the normal control group were treated with the same volume of citrate buffer. Three days after STZ was injected, the fasting blood glucose (FBG) of the rats was measured from the tail tip. The rats with FBG levels above 11.1 mmol/L were used as diabetic rats for further study (Baranwal *et al*. 2015). Fifty rats were randomly divided into five groups, with ten rats in each group. These groups were divided into DM-HD (8 mg per kg bw), DM-MD (4 mg per kg bw), DM-LD (2 mg per kg bw), normal group and diabetic model groups. The dose of 0.1 mL (soybean oil)/100 g (body weight) for the normal and diabetic model groups was administrated by gavage, and it was the same dose of soybean oil without alkylamide. The experimental rats were administered with normal feeding and drinking. Daily feed intake and weight of rats were recorded, and FBG was measured every two weeks. Test cycle was set to 28 days. Food supply was blocked for 12 h on the last day of the experimental period. The rats were then injected with ether anaesthesia and decapitated. Their blood was extracted and collected in an anticoagulant vessel (Shandong Aosite Medical Instrument Factory, Shandong, China) and then centrifuged at 2683 ***g*** 4°C for 15 min. The supernatant was preserved in −20°C. Liver, kidney and gastrocnemius were preserved at −80°C.

### Protein synthesis and protein degradation

Protein synthesis was determined by the incorporation of 14^+^-C-phenylalanine (0.05 μCi/mL) (China Institute of Atomic Energy, Beijing, China) into the skeletal muscle in unit time (Voltarelli & de Mello 2008). Weighed fresh skeletal muscle was placed in 5 mL DMEM (Hyclone, Utah, USA), incubated for 30 min with oxygen at 37°C and then incubated for 1 h in DMEM containing 14^+^-C-phenylalanine. The skeletal muscle was then washed three times with phosphate-buffered saline and homogenised. Protein was precipitated with 10% TCA; the supernatant was discarded. Protein was then dissolved with 0.5 mol/L NaOH, and 90% protein was added to the liquid scintillation count. About 10% of protein was used to determine protein concentrations by the BCA method (Nanjing Jiancheng Bioengineering Institute, Nanjing, China).

Skeletal protein catabolism was assessed by the tyrosine release rate assay of the culture medium (Li *et al*. 2016). Left skeletal muscle was weighed and immersed in Basal–Krebs–Ringer buffer (1.2 mmol/L NaCl, 4.8 mmol/L KCl, 25 mmol/L NaHCO_3_, 2.5 mmol/L CaCl_2_, 1.2 mmol/L KH_2_PO_4_, 1.2 mmol/L MgSO_4_, 5.5 mmol/L glucose, 1.0 g/L bovine serum albumin, 5 U/mL insulin and 5 mmol/L cyclohexamide at pH 7.4). The tissue was then incubated for 2 h with oxygen at 37°C. The culture medium was precipitated with TCA. Supernatant (0.5 mL) was placed in a 2 mL centrifuge tube, and then 1.0 mL 5% TCA was added and mixed with the supernatant. Subsequently, 0.75 mL nitric acid and 0.75 mL 1-nitroso-2-napthol were added and mixed. The supernatant was incubated for 30 min at 55°C and extracted by ethylene dichloride; 200 μL of supernatant was used to measure fluorescence spectrometry (Ex. 450 nm, Em. 550 nm). The final concentration of the samples was calculated by the l-tyrosine standard curve.

### Physiological and biochemical analyses

Nucleic acid content determination was conducted with reference to Leonard (Johnson & Chandler 1973). The FBG and fructosamine levels in the plasma were determined using a commercially available kit (Sichuan Maker Biotechnology Co., Ltd, Sichuan, China) on a Hitachi 7020 automatic biochemistry analyser (Hitachi High-Technologies Corp., Tokyo, Japan). Plasma insulin and the insulin-like growth factor (IGF1) were measured by RIA with the Bi-Insulin RIA kit (ERIA Diagnostics Pasteur, Marnesla Coquette, France). Blood urea nitrogen (BUN) was measured by urea enzymatic assay (Nanjing Jiancheng Bioengineering Institute, Nanjing, China) in accordance with the manufacturer’s protocols. Protein concentrations were calculated using BCA assay kits (Pierce, USA).

### RNA extraction and reverse transcription PCR (RT-PCR)

All tissues were homogenised in TRIzol Reagent (Invitrogen), and total RNA was extracted according to the RNA Extraction Kit (OMEGA, USA). The quality of extracted RNA was assessed with a NanoDrop 1000 Spectrophotometer (Thermo Scientific). Optical purity and density were 1.8 < 260/280 nm < 2.0. Reverse transcription was performed using MLV-RT (Promega).

The rat housekeeping gene β-actin was used as an internal control. Primers for the real-time qRT-PCR were designed by primer 5.0 (Table 1). Real-time qRT-PCR was performed in a CFX96 Touch Real-Time System (Bio-Rad) using SYBR Green Supermix (Bio-Rad). Amplification was conducted in a 10 µL reaction mixture containing 1 µL cDNA (100 ng/µL), 4.2 µL DEPC water, 0.3 μL forward or reverse primer (20 µmol/L) and 4.2 µL SYBR Green Supermix (Bio-Rad) in each well of a 96-well plate. The reaction procedure was 94°C for 10 s, followed by 40 cycles at 95°C for 5 s and 60°C for 40 s. Melting curve analysis was performed to confirm the specific amplification. Each expression assay was repeated three times. The CFX Manager Bio-Rad 3 software was used to process real-time quantitative PCR data after amplification. Relative expression levels of the target gene (X) were calculated in relation to the transcription levels of the actin reference gene (R) as 2^−Δ*C*_t_^, where Δ*C*_t_ = *C*^tX^−*C*^tR^. SPSS 20.0 was used to analyse the variance and significance of the results.

**Table 1:** Primer sequence and product size.

	Primer sequence		
Gene	Forward primer	Reverse primer	Product size
mTOR	ACCCAAGCCTGGGACCTCTA	GGCTGGTTGGGGTCATATGTT	156
PI3K	CTCAGGGAAAGCTGGACCAC	TGGTTCAGACGAGCTTCTGTG	184
PKB	ACTCATTCCAGACCCACGAC	CCGGTACACCACGTTCTTCT	176
Atrogin-1	CCATCAGGAGAAGTGGATCTATGTT	GCTTCCCCCAAAGTGCAGTA	84
MuRF1	ATCTGGCTTGATTCCGGACG	TGGAAGATGTCGTTGGCACA	355
FOXO1	TACGGCCAATCCAGCAT	TGGGGAGGAGAGTCAGAAGT	143
FOXO3A	CGGCTCACTTTGTCCCAGAT	TCTTGCCAGTCCCTTCGTTC	163
FOXO4A	CTGGTTTCTGGTTTCTGCTGC	TAACTGCCCTTCGACTTTCCG	93
IGF1	GGCACTCTGCTTGCTCACCTTT	CACGAATTGAAGAGCGTCCACC	91
IGF1R	CACCTGGAAGAACCGCATCATCATAA	ATCCTGCCCGTCGTATTCCGTGAC	130
InR	TCGCTCCTATGCTCTGGTGTCA	GGTTCTGGTTGTCCAAGGCGTA	110
β-Actin	TCGTACCACTGGCATTGTGAT	CGAAGTCTAGGGCAACATAGCA	233

### Western blot analysis

Skeletal muscle samples were collected from each group and homogenised in an ice-cold lysed buffer containing 2 μg/mL of protease inhibitor (Bi-Yuntian, Beijing, China). The suspensions were centrifuged at 14,000 ***g*** at 4°C for 15 min. The supernatant was collected to determine protein content through the BCA method. The proteins were separated by 10% SDS-PAGE and then transferred to a 0.22 μm PVDF membrane. The membranes were blocked with 5% defatted milk powder at 37°C for 2 h and then incubated with anti-mTOR, anti-P-mTOR (Ser2448), anti-PKB, anti-P-PKB (Ser473), anti-PI3K (p110), anti-FOXO1A, anti-FOXO3A, anti-FOXO4A, anti-atrogin-1, anti-MURF1 and anti-tubulin (Abcam) antibodies overnight at 4°C. Afterward, the membranes were washed with TBST and incubated with the corresponding species-specific secondary antibodies (Bioss, Beijing, China) at 37°C for 1 h. After the membrane was washed with TBST, the proteins were visualised through ECL (Bio-Rad) under a chemiluminescence imaging apparatus (Bio-Rad).

### Statistical analysis

Results were expressed as mean ± standard error and analysed by one-way analysis of variance. Student’s *t* test was used to detect the differences in the mean between the normal group and the diabetic rat group. The differences were considered significant when *P* < 0.05. Data were processed using SPSS 20.0 and Origin 9.0.

## Results

### HPLC analysis of alkylamides

**Figure 1:**
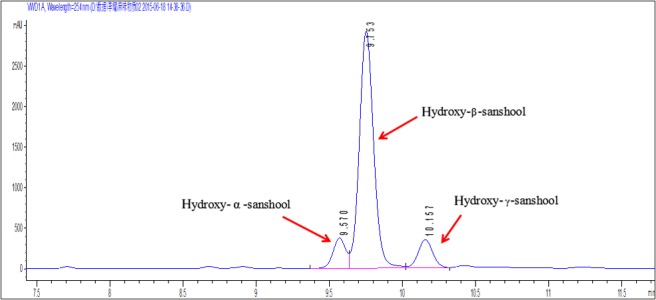
HPLC analysis of *Zanthoxylum* alkylamides. Exactly 0.05 mg alkylamides dried by nitrogen was dissolved in methanol at a volume of 5 mL and filtered through 0.45 µm microporous membrane. The samples were applied to a C18 column (4.6 mm × 250 mm, 5 µm, Agilent). Mobile phase A had 50% water, and mobile phase B had 50% methanol. The flow rate was 1 mL/min, sample volume 10 µL, column temperature 40°C and ultraviolet detection wavelength 254 nm.

The extracted alkylamides were analysed by HPLC, and the retention times of 9.570, 9.758 and 10.153 min were obtained. The relative contents of hydroxy-α-sanshool, hydroxy-β-sanshool and hydroxy-γ-sanshool were 26.48, 39.83 and 29.19%, respectively (Fig. 1), consistent with those in previous studies (Dossou *et al*. 2013, Yu 2015).

### Effect of alkylamides on the body weight and relative weight of organs

After 28 days, the weight gain of the diabetic group was significantly lower than that of the normal control group, and the weight of the alkylamide-treated group increased significantly. Compared with that of the normal group, feed intake of the STZ-induced diabetic rats and relative liver weights significantly increased, and the relative skeletal muscle weights significantly decreased. Alkylamides can significantly reduce feed intake and relative liver weights and significantly elevate the relative skeletal muscle weights (Table 2). Moreover, compared with those in the diabetic model, TP, RNA content and RNA/DNA ratio were significantly increased by alkylamides in the skeletal muscle of diabetic rats, but no significant difference was found in DNA content (Table 2).

**Table 2:** Growth performance and muscle characteristics.

	NC	DM	DM-HD	DM-MD	DM-LD
Initial weight (g)	203.47 ± 8.35^a^	205.45 ± 6.08^a^	198.50 ± 8.50^a^	208.25 ± 7.74^a^	202.88 ± 6.66^a^
Growth rate (g/4 W)	90.67 ± 7.06	15.38 ± 3.70^*a^	36.78 ± 3.91^c^	42.00 ± 4.46^c^	24.57 ± 3.35^b^
Feed intake (g/4 W)	556.70 ± 24.15	1037.76 ± 25.82^*a^	880.09 ± 30.77^b^	852.79 ± 21.29^b^	926.59 ± 26.62^b^
Skeletal muscle (%)	3.18 ± 0.29	2.19 ± 0.47^*a^	2.79 ± 0.23^b^	2.97 ± 0.59^b^	2.58 ± 0.45^c^
Liver (%)	2.91 ± 0.48	4.50 ± 0.23^*a^	3.25 ± 0.48^b^	3.46 ± 0.19^b^	3.76 ± 0.29^c^
TP (mg/g)	5.53 ± 0.14	3.94 ± 0.20^*a^	4.61 ± 0.30^b^	4.54 ± 0.77^b^	4.21 ± 0.29^b^
RNA (mg/g)	0.38 ± 0.001	0.33 ± 0.002^*a^	0.36 ± 0.001^b^	0.36 ± 0.001^b^	0.34 ± 0.001^c^
DNA (mg/g)	0.31 ± 0.005	0.31 ± 0.005	0.31 ± 0.003	0.31 ± 0.009	0.31 ± 0.011
RNA/DNA ratio	1.20 ± 0.02	1.05 ± 0.01^*a^	1.15 ± 0.01^b^	1.14 ± 0.03^b^	1.10 ± 0.04^c^

Values are the means ± s.e.m. (*n* = 10).

Means with different superscript letters are significantly different among the diabetic rats (*P* < 0.05); *means are significantly different (*P* < 0.05) from the means in the control group.

DM, diabetic rats treated with vehicle; DM-HD, diabetic rats treated with 8 mg per kg bw alkylamides; DM-LD, diabetic rats treated with 2 mg per kg bw alkylamides; DM-MD, diabetic rats treated with 4 mg per kg bw alkylamides; NC, normal rats treated with vehicle.

### Plasma parameters

The BUN level of the STZ-induced diabetic rats was markedly increased compared with that of the normal group (*P* < 0.05). Alkylamides could significantly reduce the BUN level. The BUN levels of diabetic rats administered intragastrically with high (DM-HD), middle (DM-MD) and low (DM-LD) doses of alkylamides were reduced by 25.81, 35.26 and 14.57%, respectively (Table 3). The Cr content of diabetic rats markedly increased compared with that of the normal group, and it significantly reduced compared with that of STZ-induced diabetic rats by alkylamides (Table 3).

**Table 3:** Plasma parameters.

	NC	DM	DM-HD	DM-MD	DM-LD
BUN (mmol/L)	4.95 ± 0.28	9.36 ± 0.57^*a^	7.44 ± 0.46^b^	6.92 ± 0.39^b^	8.17 ± 0.52^c^
Cr (μmol/L)	35.36 ± 1.93	46.69 ± 1.18^*a^	34.76 ± 1.38^b^	36.94 ± 1.85^b^	41.65 ± 1.73^c^
Insulin (IU/mL)	37.73 ± 1.28	14.71 ± 1.53^*a^	25.70 ± 1.42^b^	22.86 ± 1.55^b^	20.28 ± 1.35^c^
IGF1 (ng/mL)	48.14 ± 2.54	22.44 ± 2.45^*a^	35.23 ± 2.53^b^	40.77 ± 2.09^c^	32.35 ± 3.26d
TP (g/L)	64.77 ± 2.21	42.60 ± 2.13^*a^	56.68 ± 1.59^b^	54.18 ± 1.46^b^	48.69 ± 2.23^c^
ALB (g/L)	39.93 ± 1.06	16.86 ± 1.33^*a^	28.18 ± 1.44^b^	27.23 ± 1.40^b^	20.30 ± 2.10^c^
GPs (g/L)	24.84 ± 1.21	25.74 ± 0.85	28.51 ± 2.19	26.95 ± 1.01	28.39 ± 0.43
ALB/GPs	1.61 ± 0.04	0.65 ± 0.03^*a^	0.99 ± 0.12^b^	1.01 ± 0.07^b^	0.71 ± 0.08^a^

Conditions were the same as those defined in Table 2. Values are the means ± s.e.m. (*n* = 10).

Means with different superscript letters are significantly different among the diabetic rats (*P* < 0.05); *means are significantly different (*P* < 0.05) from the means in the control group.

The TP, albumin (ALB) and globulin (GPs) levels in the serum reflect the state of protein absorption and metabolism. The TP, ALB and ALB/GPs levels of the STZ-induced diabetic rats markedly decreased compared with those of the normal group. Alkylamides can significantly enhance TP, ALB and ALB/GPs levels in the serum of diabetic rats (Table 3).

**Figure 2:**
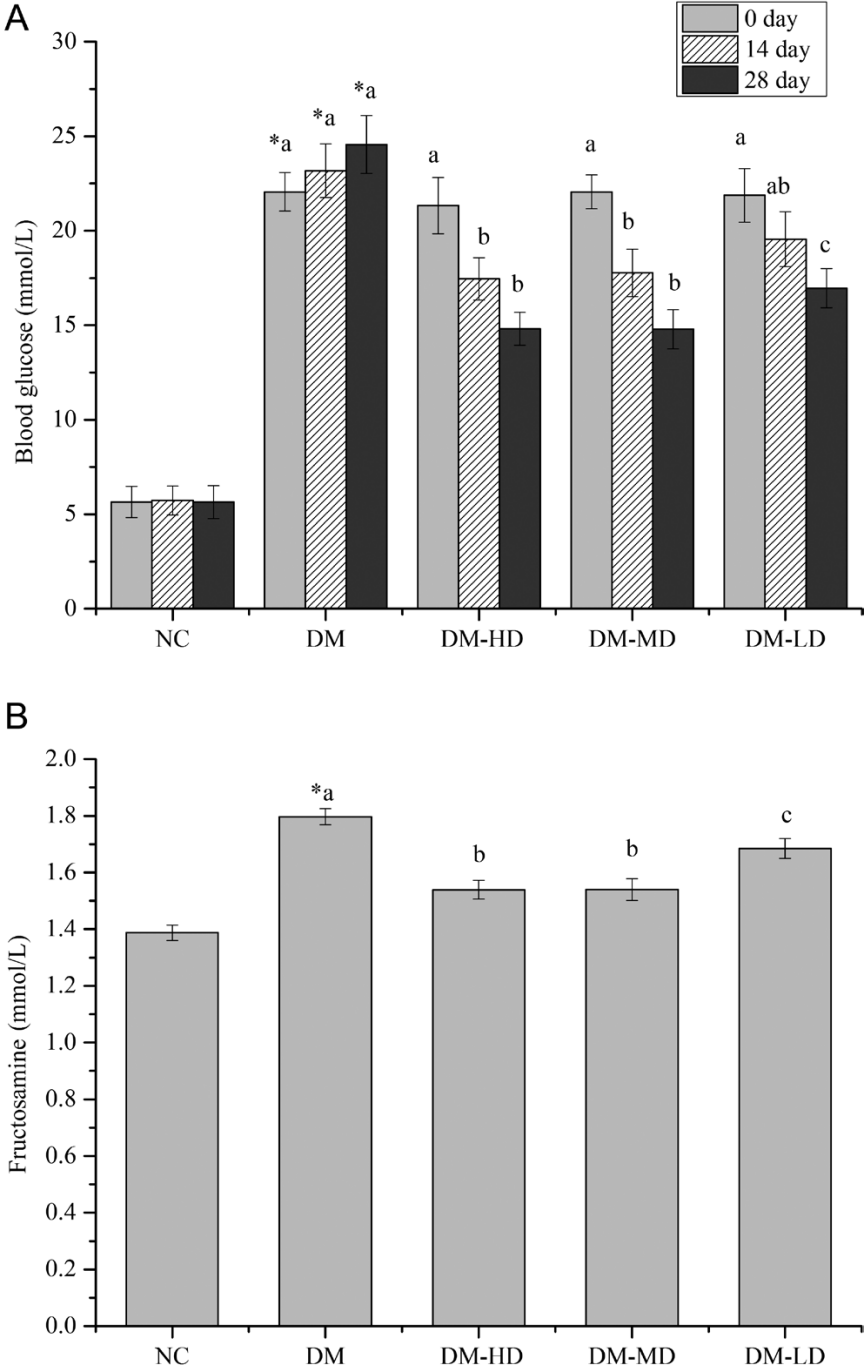
Fasting blood glucose (FBG) (A) and fructosamine levels (B) in diabetic rats treated with alkylamides. Values are the means ± s.e.m. (*n* = 10). NC, normal rats treated with vehicle; DM, diabetic rats treated with vehicle; DM-HD, diabetic rats treated with 8 mg per kg bw alkylamides; DM-MD, diabetic rats treated with 4 mg per kg bw alkylamide; DM-LD, diabetic rats treated with 2 mg per kg bw alkylamide. Means with different superscript letters are significantly different among the diabetic rats (*P* < 0.05); *means are significantly different (*P* < 0.05) from the means in the control group.

Compared with the normal group, the serum insulin content of the model group was significantly reduced by 156.49% (*P* < 0.05). With alkylamide treatment for 28 days, the serum insulin levels of diabetic rats increased by 74.71, 55.40 and 31.45% in DM-HD, DM-MD and DM-LD (*P* < 0.05), respectively. IGF1 was also significantly elevated, with DM-HD (8 mg per kg bw) and DM-MD (4 mg per kg bw) having the most significant increase (Table 3). In addition, alkylamides significantly reduced FBG and fructosamine levels in the serum of diabetic rats (Fig. 2A and B).

### Effects of alkylamides on muscle protein synthesis and protein degradation

**Figure 3:**
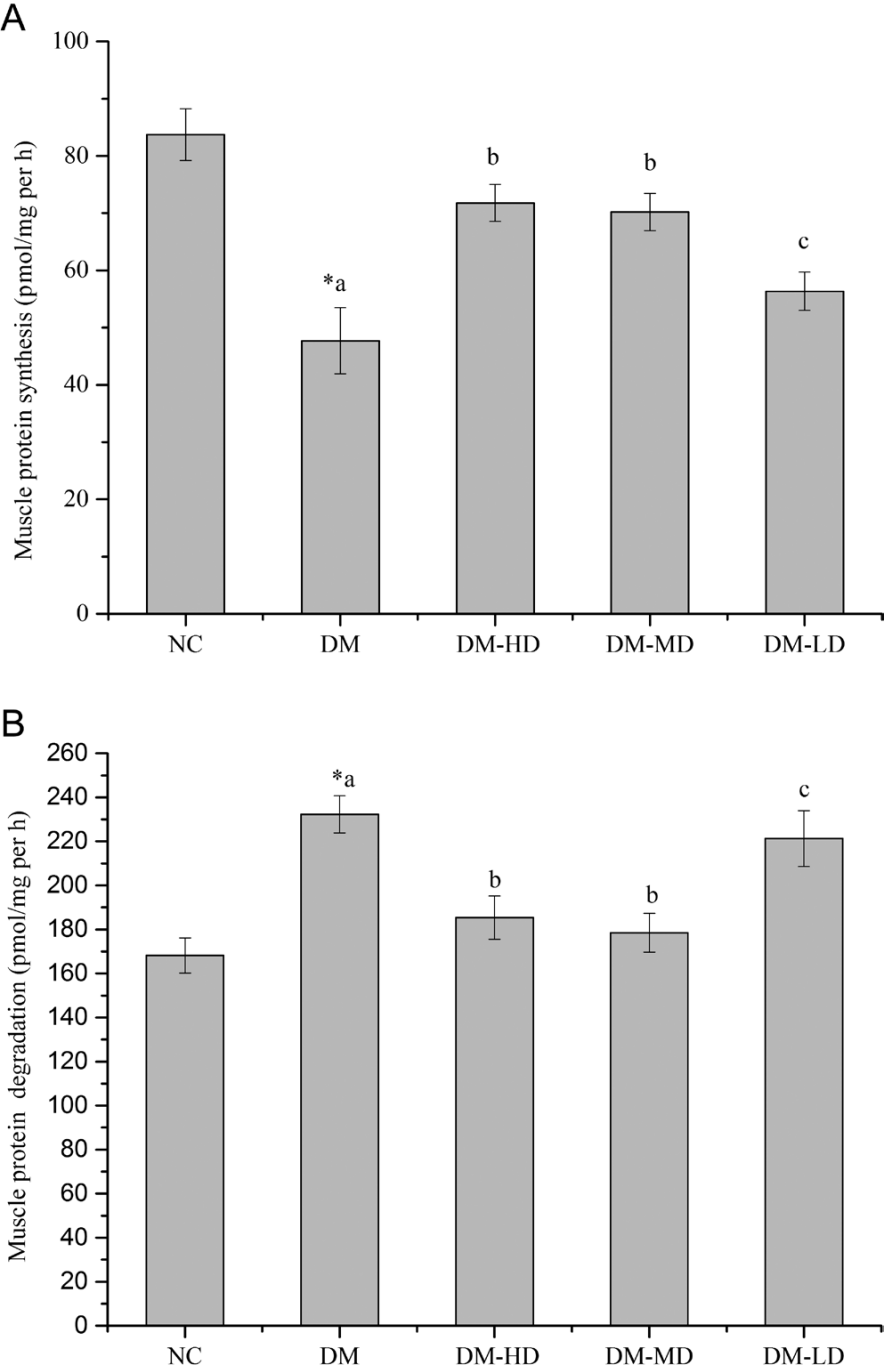
Protein synthesis and protein degradation. (A) Protein synthesis was measured from the rate of incorporation of l-[U-14C] phenylalanine into isolated, incubated skeletal muscle. (B) Protein degradation was measured as the rate of tyrosine released from isolated skeletal muscle. Values are the means ± s.e.m. (*n* = 10). Conditions were the same as those defined in Fig. 2. Means with different superscript letters are significantly different among the diabetic rats (*P* < 0.05); *means are significantly different (*P* < 0.05) from the means in the control group.

Compared with the normal findings, protein synthesis was significantly reduced and protein catabolism was significantly increased in the diabetes model (*P* < 0.05) (Fig. 3A and B). With alkylamide treatment, protein synthesis significantly increased, and the increase in high and medium doses was significantly higher than that in the low dose (Fig. 3A). Interestingly, protein catabolism was significantly reduced (Fig. 3B).

### Effect of alkylamides on the expression of genes InR, IGF1 and IGF1R

**Figure 4:**
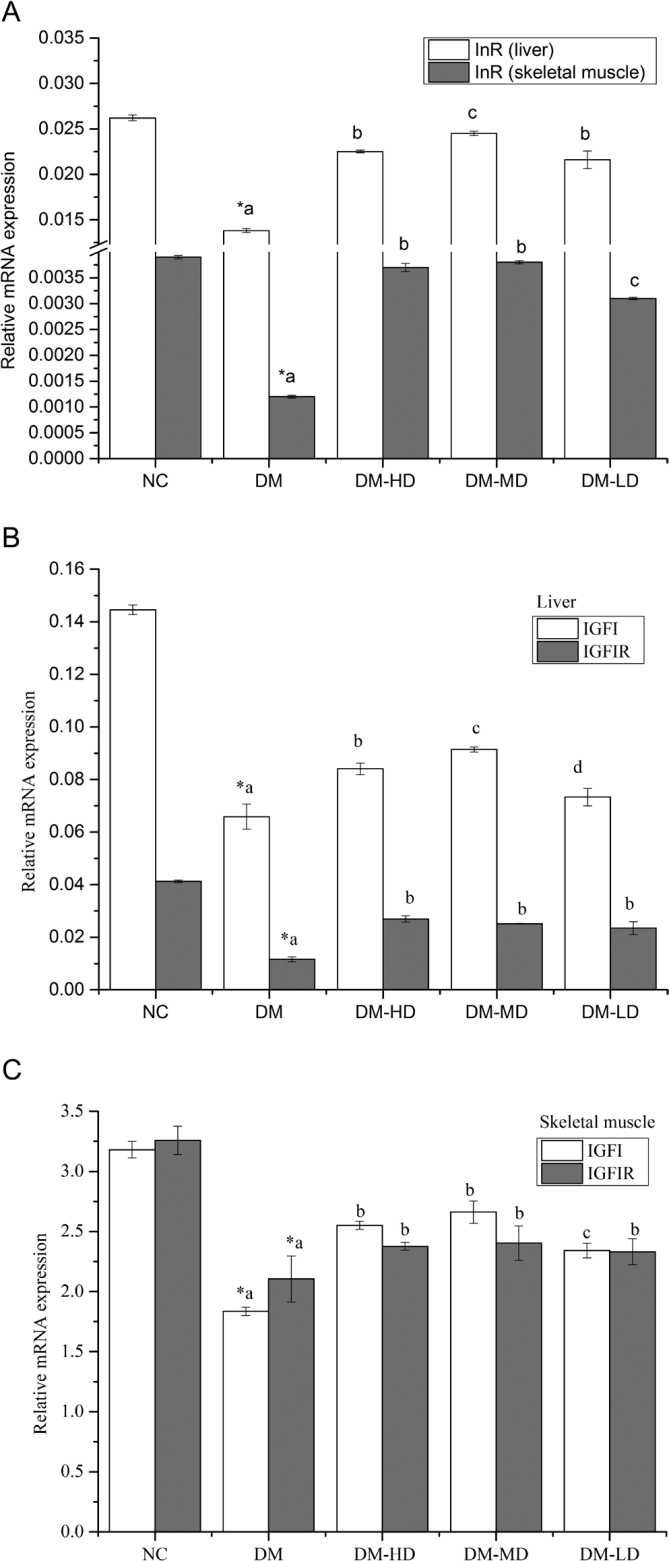
Effect of alkylamides on expression of genes InR in the liver and skeletal muscle (A), effect of alkylamides on expression of genes IGF1 and IGF1R in the liver (B), expression of genes IGF1 and IGF1R on the skeletal muscle (C). Values are the means ± s.e.m. (*n* = 10). Conditions were the same as those defined in Fig. 2. Means with different superscript letters are significantly different among the diabetic rats (*P* < 0.05); *means are significantly different (*P* < 0.05) from the means in the control group.

Compared with that of the normal group, the mRNA expressions of the insulin receptor (InR) in the liver and the skeletal muscle of the model group were significantly reduced by 89.86% and 225.00% (*P* < 0.05), respectively. With alkylamide treatment for 28 days, the mRNA expressions of InR in the liver of diabetic rats were elevated by 63.04, 77.54 and 56.52% (*P* < 0.05) and those in the skeletal muscle were elevated by 208.33, 216.67 and 158.33% (*P* < 0.05) in DM-HD, DM-MD and DM-LD, respectively (Fig. 4A).

Compared with that of the normal group, the mRNA expression of IGF1 and IGF1R in the liver and skeletal muscle of the diabetic rats markedly decreased, but it increased remarkably compared with that of the STZ-induced diabetic rats after 28 days of alkylamide treatments (Fig. 4B and C). The mRNA expressions of IGF1R in the liver of DM-HD, DM-MD and DM-LD groups did not show any significant difference (Fig. 4B).

### Skeletal muscle expression of genes related to protein synthesis

**Figure 5:**
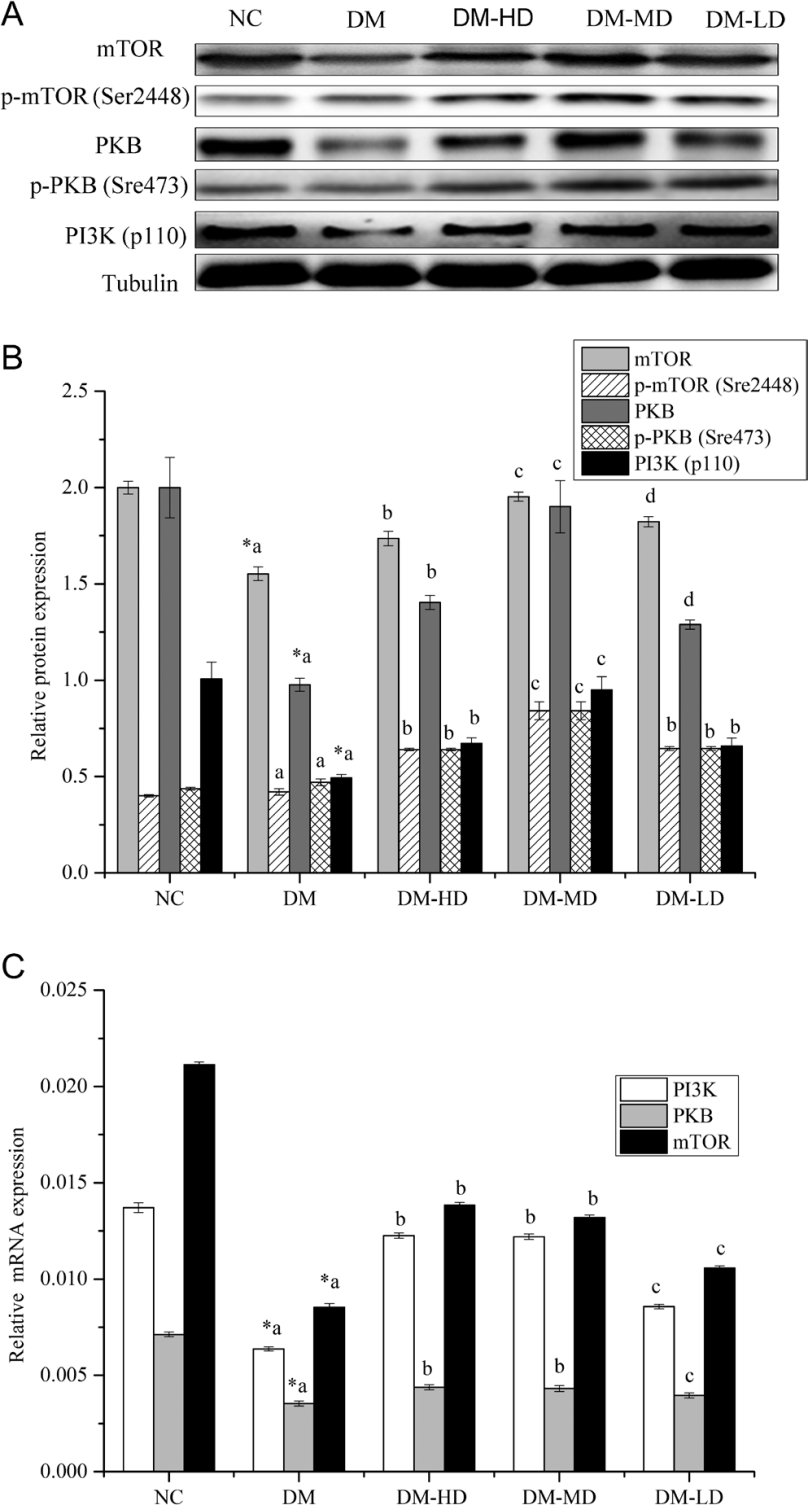
Protein synthesis protein (A and B) and gene (C) expressions in the skeletal muscle of diabetic rats treated with alkylamides. Values are the means ± s.e.m. (*n* = 10). Conditions were the same as those defined in Fig. 2. Means with different superscript letters are significantly different among the diabetic rats (*P* < 0.05); *means are significantly different (*P* < 0.05) from the means in the control group. mTOR, mammalian target of rapamycin; p-mTOR (Ser2448), the 2448 ser phosphorylation of the mTOR; PKB, protein kinase B; p-PKB (Ser473), The 473 Ser phosphorylation of the PKB; PI3K, phosphatidylinositol 3-kinase.

Compared with those in the normal control, mTOR mRNA and protein levels were reduced by 147.42% and 28.82% (*P* < 0.05), respectively; p-mTOR (Ser2448) protein levels were upregulated by 5.05% (*P* > 0.05); PKB mRNA and protein levels were reduced by 81.96% and 104.71% (*P* < 0.05), respectively; p-PKB (Ser473) protein levels were upregulated by 7.65% (*P* > 0.05); PI3K mRNA levels were reduced by 114.98% and PI3K (p110) protein levels by 103.66% (*P* < 0.05). Compared with those in the diabetic model, mTOR mRNA levels were elevated by 47.96, 47.71 and 25.55% (*P* < 0.05); mTOR protein levels were upregulated by 11.83, 25.76 and 17.46% (*P* < 0.05) and p-mTOR (Ser2448) protein levels were upregulated by 52.39, 100.21 and 53.78% (*P* < 0.05). PKB mRNA levels were elevated by 18.18, 18.06 and 10.61% (*P* < 0.05); PKB protein levels were upregulated by 43.72, 94.58 and 31.91% (*P* < 0.05) and p-PKB (Ser473) protein levels were upregulated by 36.18, 78.92 and 37.43% (*P* < 0.05). PI3K mRNA levels were elevated by 47.96, 47.71 and 25.54% (*P* < 0.05); and PI3K (p110) protein levels were upregulated by 26.47, 48.03 and 24.97% (*P* < 0.05) (Fig. 5A, B and C).

### Skeletal muscle expression of genes related to protein catabolism

**Figure 6:**
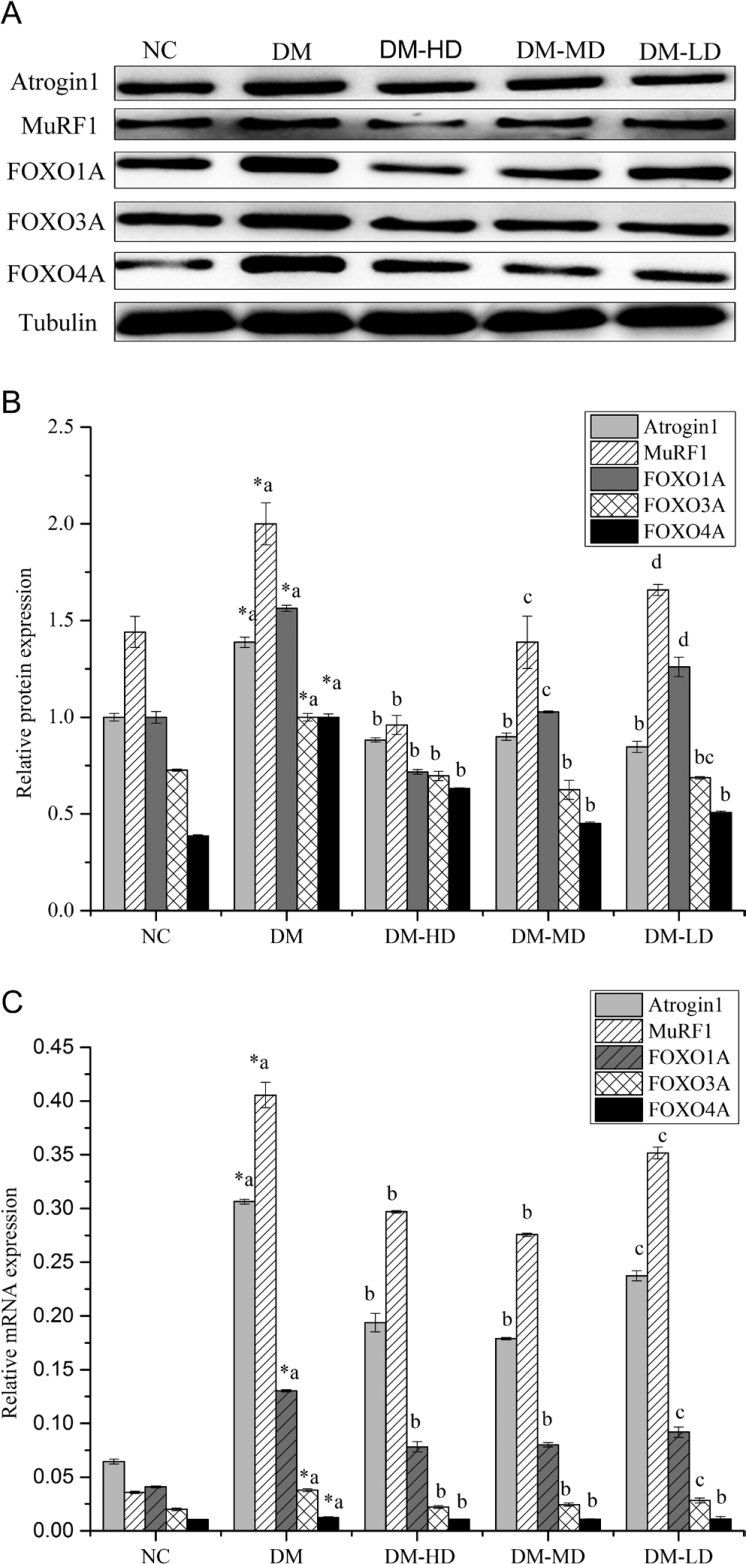
Proteolysis protein (A and B) and gene (C) expressions in the skeletal muscle of diabetic rats treated with alkylamides. Values are the means ± s.e.m. (*n* = 10). Conditions were the same as those defined in Fig. 2. Means with different superscript letters are significantly different among the diabetic rats (*P* < 0.05); *means are significantly different (*P* < 0.05) from the means in the control group. Atrogin-1, bxmuscle atrophy F-box; MuRF1, muscle ring finger 1; FOXO, forkhead box-O, the FOXO transcription factors consist of FOXO1A, FOXO3A and FOXO4A.

Compared with the normal control, muscle atrophy F-box (MAFbx/atrogin-1) mRNA and protein levels were increased by 78.94% and 27.95% (*P* < 0.05); muscle ring finger 1 (MURF1) mRNA and protein levels were increased by 91.16% and 27.97%; FOXOA1 mRNA and protein levels were increased by 68.60% and 36.07%; FOXOA3 mRNA and protein levels were increased by 46.94% and 27.34% and FOXOA4 mRNA and protein levels were increased by 16.13% and 61.45%, respectively (Fig. 6A, B and C). Compared with the diabetic model, groups that received alkylamide treatment for 28 days downregulated atrogin-1 mRNA levels by 58.20, 71.32 and 53.73% and atrogin-1 protein expression levels were upregulated by 57.27, 54.37 and 63.86% in DM-HD, DM-MD and DM-LD (*P* < 0.05), respectively. MURF1 mRNA levels were downregulated by 36.56, 47.13 and 15.36%, and MURF1 protein expression levels were upregulated by 108.16, 44.14 and 20.66% in DM-HD, DM-MD and DM-LD (*P* < 0.05), respectively. FOXOA1 mRNA levels were downregulated by 66.78, 62.78 and 41.79%, and FOXOA1 protein expression levels were upregulated by 118.15, 52.25 and 24.08% in DM-HD, DM-MD and DM-LD (*P* < 0.05), respectively. FOXOA3 mRNA levels were downregulated by 70.60, 54.83 and 33.94%, and FOXOA3 protein expression levels were upregulated by 43.31, 59.97 and 45.48% in DM-HD, DM-MD and DM-LD (*P* < 0.05), respectively. FOXOA4 mRNA levels were downregulated by 16.00, 13.87 and 12.83%, and FOXOA4 protein expression levels were upregulated by 86.91, 121.39 and 96.85% in DM-HD, DM-MD and DM-LD (*P* < 0.05), respectively (Fig. 6A, B and C).

## Discussion

Diabetes mellitus is a metabolic disorder. Although many thorough studies have been conducted on type 2 diabetes mellitus (T2DM), the incidence of type 1 diabetes (T1DM), which has extremely serious complications, is increasing yearly (Tierney *et al*. 2012). Therefore, in recent years, the pathogenesis of T1DM has been examined intensely in research. STZ is widely used in the construction of the diabetes model experiment because it can specifically destruct pancreatic beta cells, which cause T1DM (Bortolin *et al*. 2015, Carlos *et al*. 2015, Lin & Sun 2015). In this experiment, the FBG levels of the normal control group were maintained within the normal range (4–6 mmol/L) throughout the feeding period. The diabetic group always achieved an FBG greater than 11 mmol/L, thus showing that the model was constructed successfully and stably. The FBG and fructosamine levels of the rats in the alkylamide-treated groups significantly decreased after two weeks of the experiment (Fig. 2A and B). This finding is consistent with that in previous studies (You *et al*. 2015).

To a certain extent, as an important biochemical index of animals, TP reflects the level of protein deposition (Friesen *et al*. 1992). The BUN level is a reflection of protein metabolism status and can be used as an indicator of nitrogen utilisation and protein deposition. Decreased BUN can increase nitrogen deposition and protein synthesis (Chikhou *et al*. 1993, Newell 1999, Wang et al. 2011 Zhang *et al*. 2012). Results showed that protein contents in the serum and skeletal muscle significantly increased and that the BUN content was significantly decreased by alkylamides in diabetic rats (Tables 2 and 3). These findings reveal the increase in protein deposition of diabetic rats. Messenger RNA and transporter RNA are involved in protein synthesis. Once the protein synthesis rate is accelerated, RNA content increases. Thus, RNA content is a sensitive index for measuring the biological growth rate. The number of organelles in cells is constant, and DNA content is not sensitive to the change in environmental conditions (Dorch *et al*. 1983). The RNA/DNA ratio was not affected by the number and cell size in the sample; thus, estimating the growth state is more accurate than the single RNA content (Laimek *et al*. 2008). Results show that TP content, RNA content and RNA/DNA ratio of skeletal muscle were increased by alkylamides in diabetic rats. Conversely, DNA content did not significantly change and the apparent relative muscle skeletal weight increased (Tables 2 and 3). At the same time, alkylamides accelerated skeletal muscle protein synthesis in diabetic rats in the *in vitro* experiment (Fig. 2A). These results imply that alkylamides can accelerate protein synthesis and promote skeletal muscle protein deposition in diabetic rats.

Diabetes not only increases blood glucose and lipid levels but also causes protein metabolism disorder (Michaelides *et al*. 2016). Consumptive disease usually decreases total serum protein and ALB (Hu *et al*. 2004). The protein consumption of diabetic rats was higher than that of the normal control group with alkylamide treatment, and the TP and ALB consumption was reduced (Table 3). ALB/GPs <1 commonly exist in nephrotic syndrome (Zhon 2015). ALB/GPs >1.0 in the normal control, ALB/GPs <1.0 in the diabetic model and the BUN and Cr in the diabetic model were significantly higher than those in the normal group. These values show that the diabetic rats suffered from a complicated kidney disease caused by protein metabolism disorders. With alkylamide treatment, BUN and Cr content significantly decreased in the serum, and the ratio of ALB/GPs was higher than that of the model group (Table 3). *In vitro* skeletal muscle culture also indicated that alkylamides could significantly reduce muscle protein catabolism (Fig. 2B). This finding reveals that alkylamides can regulate protein metabolism. The mechanism may be that alkylamides repair damaged islet cells and tissues and promote the secretion of insulin (You *et al*. 2015). With alkylamide treatment, plasma insulin and IGF1 level of the alkylamide-treated group were significantly elevated (Table 3). Insulin can promote protein synthesis and inhibit protein decomposition (Michaelides *et al*. 2013). Increased insulin and IGF1 contents in the serum can enhance the protein metabolism of the body. The parameter results indicate that alkylamides can ameliorate protein metabolism disturbance in STZ-induced diabetic rats. In addition, IGF1 can stimulate skeletal muscle development through the autocrine and paracrine pathways (Sacheck *et al*. 2004). Insulin and IGF1 functions are performed mainly by binding to their respective receptor InR and IGF1R (Brisson & Barton 2012, Shen *et al*. 2015). The mRNA expression of InR, IGF1 and IGF1R was significantly increased by alkylamides in the liver and skeletal muscle of the diabetic rats (Fig. 4A, B and C). Alkylamides may ameliorate protein metabolism disturbance through the mediation of insulin and IGF1.

In the past few years, studies were conducted on insulin and IGF1 signalling pathways leading to translation initiation, which regulates the changes in protein synthesis. mTOR can be activated by amino acids and insulin stimulation (Wang & Proud 2010, Liu *et al*. 2012). The main stage of the entire process of cellular protein synthesis is the translation of proteins, and the translation initiation process determines the translation speed of mRNA. mTOR plays a key role in process of IGF1-, insulin- and amino acid-regulating translation initiation. In the regulatory pathways, IGF1 binds with IGF1R to form the IGF1R/IR complex; multi-tyrosine residues of phosphorylated IGF1R/IR are activated; activated IGF1R/IR recruits the p85 subunit of PI3K through the phosphorylated substrate protein and transmits a signal to the P110 subunit to trigger the activation of PI3K; activated PI3K catalyses the tertiary phosphorylation of PI-4-P, PI-4 and 5-P2 to create PI3, 4-P2 and PI3, 4 and 5-P3; and the downstream molecules PKB convene to the cytomembrane. PI-3, 4 and 5-P3 can activate phosphatidylcholine-dependent kinase-1 (PDK-1), thus inducing the 308 Thr phosphorylation of the PKB in the presence of PDK-2. The 473 Ser phosphorylation of the PKB (phosphorylated PKB) activates mTOR through the activation of Rheb (the Ras homolog enriched in the brain), which is the upstream factor of mTOR (Fan *et al*. 2004, Kang & Cho 2015, Meena & Kanwar 2015, Sun *et al*. 2016). Moreover, amino acid can be used as a signal molecule to directly act on the mTOR for activation (Hara *et al*. 1998, Kimball & Jefferson 2006, Dukes *et al*. 2015, Trevizani *et al*. 2015). Activated mTOR improves protein synthesis through the direct phosphorylation of the eukaryotic translation initiation factor 4E-binding protein 1 (4EBP1) and ribosome protein S6 kinase 1 (S6K1). The phosphorylation of 4EBP1 inhibits the binding to the cap-binding protein, making it and the cap structure dependent on the translation initiation required by the eukaryotic translation initiation factor complex formation. The S6K1 activation of various effector proteins increases the mRNA expression in the process of translation initiation and elongation (Karlsson *et al*. 2013, 2015, Morita *et al*. 2013). The mRNA and protein expression levels of mTOR, PKB and PI3K in the skeletal muscle of the diabetic rats decreased remarkably compared with those of the normal group (Fig. 5A, B and C). Alkylamides significantly increased the mRNA expressions of mTOR, PKB and PI3K of DM-HD and DM-MD, which were better than those of DM-LD (Fig. 5C). Furthermore, the protein expressions of PI3K (p110), phosphorylated mTOR (ser2448) and phosphorylated PKB (ser437) of the diabetic rats increased as shown by Western blot (Fig. 5A and B). The results indicated that alkylamides could reduce the risk of protein synthesis reduction in STZ-induced diabetics. The signal pathway was Ins/IGF1→PI3K→PKB→mTOR.

UPP was the most vital and selective protein degradation pathway in mammalian cells (Nielsen *et al*. 1994, Chen *et al*. 2012). Under the joint actions of ubiquitin (Ub)-activating enzyme (E1), Ub-carrier protein (E2) and Ub-ligase (E3), ubiquitin binded with the target protein, ubiquitinated proteins were degraded into amino acids and a few small oligopeptides by 26S proteasome (Lu *et al*. 2013). The connection speed of Ub binding with the target protein was considered a rate-limiting step of UPP pathway, which was mediated by muscle atrophy F-box (atrogin-1/MAFbx) and MURF1. The activation of FOXO proteins by dephosphorylation was transferred from the cytoplasm into the nucleus to promote the transcription of atrogin-1/MAFbx and MURF1 (Brownawell *et al*. 2001, Zhang *et al*. 2002, Stitt *et al*. 2004, Stefanetti *et al*. 2015). This experiment showed that the mRNA expressions of atrogin-1, MURF1, FOXO1A, FOXO3A and FOXO4A of the diabetic rats markedly increased compared with those of the normal group. Consequently, the insufficient secretion of insulin in the diabetic rats not only caused carbohydrate and lipid metabolism disorders but also led to protein metabolism disorders, including decreased protein synthesis and strengthened protein catabolism. In the alkylamide treatments, the mRNA and protein expression levels of atrogin-1 and MURF1 significantly decreased compared with those of the diabetic rats (Fig. 6A, B and C).

The main FOXO transcription factors consist of FOXO1A, FOXO3A and FOXO4A, which act as the upstream signals of muscle-specific E3 ligase, atrogin-1 and MURF1 (Pond *et al*. 2014). The mRNA and protein expression levels of FOXO1A, FOXO3A and FOXO4A exhibit similar responses to alkylamide treatments. Therefore, the correlation between the levels of FOXO activation and the transcription of its downstream elements (atrogin-1/MAFbx and MURF1) can be clarified (Fig. 6A, B and C) (Tesseraud *et al*. 2009). In view of the molecular signal transduction pathway, mTOR and MURF1/MAFbx are regulated by the upstream factor PKB. In the case of muscle atrophy, the declining activity of PI3K decreases the phosphorylation level of PKB. The inactivation of mTOR decreased protein synthesis. Conversely, FOXO entered the nucleus and initiated the transcription of MURF1 and MAFbx, thus increasing the decomposition of protein. Ultimately, this leads to the loss and atrophy of skeletal muscle, whereas the mechanism of skeletal muscle hypertrophy was opposite (Sandri *et al*. 2004, Kimball & Jefferson 2006, Shenkman *et al*. 2015). Therefore, mTOR plays a vital role in the process of protein synthesis and decomposition.

## Conclusion

The alkylamide regulation mechanism of protein metabolism in diabetic rats increases the secretion of insulin, enhances protein synthesis by upregulating the mRNA and protein expression levels of PI3K, PKB and mTOR in skeletal muscles and reduces protein catabolism by downregulating the mRNA and protein expression levels of atrogin-1/MAFbx, MURF1 and FOXO in skeletal muscles. This study illustrates that alkylamides may increase protein synthesis through the Ins/IGF1→PI3K→PKB→mTOR pathway and reduce protein catabolism through the UPP pathway in skeletal muscles in STZ diabetic rats. Unfortunately, the mTOR pathway is affected by amino acid and energy levels; thus, this topic should be explored further. As hydroxy-α-sanshoo, hydroxy-β-sanshool and hydroxy-γ-sanshool mixtures were used in the experiments, more studies should be conducted to isolate their individual components and examine their efficacies separately. The optimal dosage and treatment effects of alkylamides as well as the effect of alkylamides on the treatment of T2DM should be further investigated.

## Declaration of interest

The authors declare that there is no conflict of interest that could be perceived as prejudicing the impartiality of the research reported.

## Funding

This work was supported by the National Natural Science Foundation of China (NSFC 31371834), the Basis and Frontline of Research Project in Chongqing of China (cstc2014jcyjA10073) and Southwest University Courtyard Level Projects (2015).

## Author contribution statement

T Y Ren and J Q Kan conceived and designed the study; T Y Ren and Y P Zhu were involved in the study concept and design and in drafting the manuscript; X J Xia, Y B Ding and Jing Guo collected the data.

## References

[bib1] Baranwal A Mirbolooki MR Mukherjee J 2015 Initial assessment of β3-adrenoceptor-activated brown adipose tissue in streptozotocin-induced type 1 diabetes rodent model using [18F] fluorodeoxyglucose positron emission tomography/computed tomography. Molecular Imaging 14 22–33. 26637263 10.2310/7290.2015.00028

[bib2] Batool F Sabir SM Rocha JBT Shah AH Saify ZS Ahmed SD 2010 Evaluation of antioxidant and free radical scavenging activities of fruit extract from *Zanthoxylum* alatum: a commonly used spice from Pakistan. Pakistan Journal of Botany 42 4299–4311.

[bib3] Bortolin RH da Graça Azevedo Abreu BJ Abbott Galvão Ururahy M Costa de Souza KS Bezerra JF Loureiro MB da Silva FS Marques DE Batista AA Oliveira G 2015 Protection against T1DM-induced bone loss by zinc supplementation: biomechanical, histomorphometric, and molecular analyses in STZ-induced diabetic rats. PLoS ONE 10 e0125349 (10.1371/journal.pone.0125349) 25933189 PMC4416905

[bib4] Brisson BK Barton ER 2012 Insulin-like growth factor-I E-peptide activity is dependent on the IGF-I receptor. PLoS ONE 7 e45588 (10.1371/journal.pone.0045588) 23029120 PMC3448668

[bib5] Brownawell AM Kops GJ Macara IG Burgering BM 2001 Inhibition of nuclear import by protein kinase B (Akt) regulates the subcellular distribution and activity of the forkhead transcription factor AFX. Molecular and Cellular Biology 21 3534–3546. (10.1128/MCB.21.10.3534-3546.2001) 11313479 PMC100275

[bib6] Carlos D Yaochite JN Rocha FA Toso VD Malmegrim KC Ramos SG Jamur MC Oliver C Camara NO Andrade MV 2015 Mast cells control insulitis and increase Treg cells to confer protection against STZ-induced type 1 diabetes in mice. European Journal of Immunology 45 2873–2885. (10.1002/eji.201545498) 26234742

[bib7] Chen K Cheng HH Zhou RJ 2012 Molecular mechanisms and functions of autophagy and the ubiquitin–proteasome pathway. Hereditas 34 5–18. (10.3724/SP.J.1005.2012.00005) 22306868

[bib8] Chikhou FH Moloney AP Allen P Quirke JF Austin FH Roche JF 1993 Long-term effects of cimaterol in Friesian steers: I. Growth, feed efficiency, and selected carcass traits. Journal of Animal Science 71 906–913. 8097508 10.2527/1993.714906x

[bib9] Chou ST Chan HH Peng HY Liou MJ Wu TS 2011 Isolation of substances with antiproliferative andapoptosis-inducing activities against leukemia cells from the leaves of Zanthoxylum ailanthoides Sieb. & Zucc. Phytomedicine 18 344–348. (10.1016/j.phymed.2010.08.018) 21036577

[bib10] Dorch Q Roberts TL Clayton JR Ahmed SI 1983 RNA/DNA ratios and DNA concentration as indicators of growth rate in planktonic marine organisms. Marine Ecology Progress 13 61–71. (10.3354/meps013061)

[bib11] Dossou KS Devkota KP Morton C Egan JM Lu G Beutler JA Moaddel R 2013 Identification of CB1/CB2 ligands from Zanthoxylum bungeanum. Journal of Natural Products 76 2060–2064. (10.1021/np400478c) 24175626 PMC8385540

[bib12] Dukes A Davis C El Refaey M Upadhyay S Mork S Arounleut P Johnson MH Hill WD Isales CM Hamrick MW 2015 The aromatic amino acid tryptophan stimulates skeletal muscle IGF1/p70s6k/mTor signaling in vivo and the expression of myogenic genes in vitro. Nutrition 31 1018–1024. (10.1016/j.nut.2015.02.011) 26059377 PMC4465076

[bib13] Fan MZ Matthews JC Etienne NM Stoll B Lackeyram D Burrin DG 2004 Expression of apical membrane l-glutamate transporters in neonatal porcine epithelial cells along the small intestinal crypt-villus axis. American Journal of Physiology: Gastrointestinal and Liver Physiology 287 G385–G398. 10.1152/ajpgi.00232.2003 15044176

[bib14] Fogg VC Lanning NJ Mackeigan JP 2011 Mitochondria in cancer: at the crossroads of life and death. Chinese Journal of Cancer 30 526–539. (10.5732/cjc.011.10018) 21801601 PMC3336361

[bib15] Friesen OD Guenter W Marquardt RR Rotter BA 1992 The effect of enzyme supplementation on the apparent metabolizable energy and nutrient digestibilities of wheat, barley, oats, and rye for the young broiler chick. Poultry Science 71 1710–1721. (10.3382/ps.0711710) 1454688

[bib16] Guo T Deng YX Xie H Yao CY Cai CC Pan SL Wang YL 2011 Antinociceptive and anti-inflammatory activities of ethyl acetate fraction from Zanthoxylum armatum in mice. Fitoterapia 82 347–351. (10.1016/j.fitote.2010.11.004) 21059381

[bib17] Hara K Yonezawa K Weng QP Kozlowski MT Belham C Avruch J 1998 Amino acid sufficiency and mTOR regulate p70 S6 kinase and eIF-4E BP1 through a common effector mechanism. Journal of Biological Chemistry 273 14484–14494. (10.1074/jbc.273.23.14484) 9603962

[bib18] Hu F-L Xuan H-Z Zhan Y-F 2004 Effect of proplis on protein metabolism in diabetes mellitus SD rats. Bee Technology 1 2–3.

[bib19] Inzucchi SE 2002 Oral antihyperglycemic therapy for type 2 diabetes: scientific review. JAMA 287 360–372. (10.1001/jama.287.3.360) 11790216

[bib20] Johnson LR Chandler AM 1973 RNA and DNA of gastric and duodenal mucosa in antrectomized and gastrin-treated rats. American Journal of Physiology 224 937–940. 4698810 10.1152/ajplegacy.1973.224.4.937

[bib21] Kang EB Cho JY 2015 Effect of treadmill exercise on PI3K/AKT/mTOR, autophagy, and Tau hyperphosphorylation in the cerebral cortex of NSE/htau23 transgenic mice. Journal of Exercise Nutrition and Biochemistry 19 199–209. (10.5717/jenb.2015.15090806) 26527331 PMC4624121

[bib22] Karlsson E Pérez-Tenorio G Amin R Bostner J Skoog L Fornander T Sgroi DC Nordenskjöld B Hallbeck AL Stål O 2013 The mTOR effectors 4EBP1 and S6K2 are frequently coexpressed, and associated with a poor prognosis and endocrine resistance in breast cancer: a retrospective study including patients from the randomised Stockholm tmoxifen trials. Breast Cancer Research 15 R96 (10.1186/bcr3557) 24131622 PMC3978839

[bib23] Karlsson E Magić I Bostner J Hallbeck A-L Stal O Magic I Dyrager C Lundstrom P Lysholm F 2015 Revealing different roles of the mTOR-targets S6K1 and S6K2 in breast cancer by expression profiling and structural analysis. PLoS ONE 10 e0145013 (10.1371/journal.pone.0145013) 26698305 PMC4689523

[bib24] Kimball SR Jefferson LS 2006 New functions for amino acids: effects on gene transcription and translation. American Journal of Clinical Nutrition 83 500S–507S. 16470021 10.1093/ajcn/83.2.500S

[bib25] Laimek P Clark S Stewart M 2008 The presence of GABA in gastropod mucus and its role in inducing larval settlement. Journal of Experimental Marine Biology and Ecology 354 182–191. (10.1016/j.jembe.2007.11.003)

[bib26] Li X Jung JJ Nie L Razavian M Zhang J Sadeghi MM Li X Jung J-J Nie L Razavian M 2016 The neuropilin-like protein ESDN regulates insulin signaling and sensitivity. American Journal of Physiology: Heart and Circulatory Physiology 310 H1184–H1193. (10.1152/ajpheart.00782.2015) 26921437 PMC4867389

[bib27] Lim HJ Wang X Crowe P Goldstein D Yang JL 2016 Targeting the PI3K/PTEN/AKT/mTOR pathway in treatment of sarcoma cell lines. Anticancer Research 36 5765–5771. (10.21873/anticanres.11160) 27793898

[bib28] Lin Y Sun Z 2015 Antiaging gene klotho attenuates pancreatic β-cell apoptosis in type 1 diabetes. Diabetes 64 4298–4311. (10.2337/db15-0066) 26340932 PMC4657580

[bib29] Liu X Yuan H Niu Y Niu W Fu L 2012 The role of AMPK/mTOR/S6K1 signaling axis in mediating the physiological process of exercise-induced insulin sensitization in skeletal muscle of C57BL/6 mice. Biochimica et Biophysica Acta 1822 1716–1726. (10.1016/j.bbadis.2012.07.008) 22846606

[bib30] Lu L Li D He FC 2013 Bioinformatics advances in protein ubiquitination. Hereditas 35 17–26. (10.3724/SP.J.1005.2013.00017) 23357261

[bib31] Meena KR Kanwar SS 2015 Lipopeptides as the antifungal and antibacterial agents: applications in food safety and therapeutics. BioMed Research International 2015 1–9. (10.1155/2015/473050) PMC4303012 25632392

[bib32] Michaelides A Raby C Wood M Farr K Toro-Ramos T 2016 Weight loss efficacy of a novel mobile Diabetes Prevention Program delivery platform with human coaching. BMJ Open Diabetes Research and Care 4 e000264 (10.1136/bmjdrc-2016-000264) PMC5020857 27651911

[bib33] Morita M Gravel SP Chénard V Sikström K Zheng L Alain T Gandin V Avizonis D Arguello M Zakaria C 2013 mTORC1 controls mitochondrial activity and biogenesis through 4E-BP-dependent translational regulation. Cell Metabolism 18 698–711. (10.1016/j.cmet.2013.10.001) 24206664

[bib34] Newell CW 1999 Nutrient floe and manure management in the mink industry. PhD thesis. Halifax, NS, Canada: Truro and Dalhousie University.

[bib35] Nielsen K Kondrup J Elsner P Juul A Jensen ES 1994 Casein and soya-bean protein have different effects on whole body protein turnover at the same nitrogen balance. British Journal of Nutrition 72 69–81. (10.1079/BJN19940010) 7918330

[bib36] Oh KJ Han HS Kim MJ Koo SH 2013 Transcriptional regulators of hepatic gluconeogenesis. Archives of Pharmacal Research 36 189–200. (10.1007/s12272-013-0018-5) 23361586

[bib37] Pond AL Nedele C Wang WH Wang X Walther C Jaeger C Bradley KS Du H Fujita N Hockerman GH 2014 The mERG1a channel modulates skeletal muscle MuRF1, but not MAFbx, expression. Muscle and Nerve 49 378–388. (10.1002/mus.23924) 23761265 PMC4056345

[bib38] Qiao Z Xie K Liu K Li G 2014 Decreased neuronal bursting and phase synchrony in the hippocampus of streptozotocin diabetic rats. Journal of Diabetes Research 2014 626108 (10.1155/2014/626108) 25093193 PMC4100371

[bib39] Sacheck JM Ohtsuka A McLary SC Goldberg AL 2004 IGF-I stimulates muscle growth by suppressing protein breakdown and expression of atrophy-related ubiquitin ligases, atrogin-1 and MuRF1. American Journal of Physiology: Endocrinology and Metabolism 287 E591–E601. (10.1152/ajpendo.00073.2004) 15100091

[bib40] Sandri M Sandri C Gilbert A Skurk C Calabria E Picard A Walsh K Schiaffino S Lecker SH Goldberg AL 2004 Foxo transcription factors induce the atrophy-related ubiquitin ligase atrogin-1 and cause skeletal muscle atrophy. Cell 117 399–412. (10.1016/S0092-8674(04)00400-3) 15109499 PMC3619734

[bib41] Shen X Xi G Wai C Clemmons DR 2015 The coordinate cellular response to insulin-like growth factor-I (IGF-I) and insulin-like growth factor-binding protein-2 (IGFBP-2) is regulated through vimentin binding to receptor tyrosine phosphatase β (RPTPβ). Journal of Biological Chemistry 290 11578–11590. (10.1074/jbc.M114.620237) 25787077 PMC4416861

[bib42] Shenkman BS Belova SP Lomonosova YN Kostrominova TY Nemirovskaya TL 2015 Calpain-dependent regulation of the skeletal muscle atrophy following unloading. Archives of Biochemistry and Biophysics 584 36–41. (10.1016/j.abb.2015.07.011) 26297661

[bib43] Stefanetti RJ Lamon S Wallace M Vendelbo MH Russell AP Vissing K 2015 Regulation of ubiquitin–proteasome pathway molecular markers in response to endurance and resistance exercise and training. Pflügers Archiv 467 1523–1537.(10.1007/s00424-014-1587-y) 25104573

[bib44] Stitt TN Drujan D Clarke BA Panaro F Timofeyva Y Kline WO Gonzalez M Yancopoulos GD Glass DJ 2004 The IGF-1/PI3K/Akt pathway prevents expression of muscle atrophy-induced ubiquitin ligases by inhibiting FOXO transcription factors. Molecular Cell 14 395–403. (10.1016/S1097-2765(04)00211-4) 15125842

[bib45] Sun P Wei S Wei X Wang J Zhang Y Qiao M Wu J 2016 Anger emotional stress influences VEGF/VEGFR2 and its induced PI3K/AKT/mTOR signaling pathway. Neural Plasticity 2016 4129015 (10.1155/2016/6720420) 27057362 PMC4769761

[bib46] Tesseraud S Bouvarel I Collin A Audouin E Crochet S Seiliez I Leterrier C 2009 Daily variations in dietary lysine content alter the expression of genes related toprotein catabolism in chicken pectoralis major muscle. Journal of Nutrition 139 38–43. (10.3945/jn.108.095752) 19056657

[bib47] Tierney LM Papadakis MA 2012 *Current Medical Diagnosis and Treatment*, International Editioned pp 1203–1215. New York, NY, USA: Lange Medical Books/Mc Graw-Hill.

[bib48] Trevizani GA Peçanha T Nasario-Junior O Vianna JM Silva LP Nadal J 2015 Cardiac autonomic responses after resistance exercise in treated hypertensive subjects. Frontiers in Physiology 6 1–6. (10.3389/fphys.2015.00258) 26441677 PMC4584945

[bib49] Voltarelli FA de Mello MA 2008 Spirulina enhanced the skeletal muscle protein in growing rats. European Journal of Nutrition 47 393–400. (10.1007/s00394-008-0740-9) 18807105

[bib50] Wang X Proud CG 2010 mTORC1 signaling: what we still don’t know. Journal of Molecular Cell Biology 3 206–220. (10.1093/jmcb/mjq038) 21138990

[bib51] Wang M Chen J Kong L Ma T Zou X 2011 Effects of creatine pyruvate on lipid and protein metabolism in broiler chickens. Journal of Nanjing Agricultural University 1 89–94.

[bib52] Xia L You J Li G Sun Z Suo Y 2011 Compositional and antioxidant activity analysis of Zanthoxylum bungeanum seed oil obtained by supercritical CO2 fluid extraction. Journal of the American Oil Chemists Society 88 23–32. (10.1007/s11746-010-1644-4)

[bib53] You Y Ren T Zhang S Shirima GG 2015 Hypoglycemic effects of Zanthoxylum alkylamides by enhancing glucose metabolism and ameliorating pancreatic dysfunction in streptozotocin-induced diabetic rats. Food and Function 6 3144–3154. (10.1039/C5FO00432B) 26222710

[bib54] Yu Y 2015 PhD thesis. Chongqing, China: Southwest University.

[bib55] Zhang X Gan L Pan H Guo S He X Olson ST Mesecar A Adam S Unterman TG 2002 Phosphorylation of serine 256 suppresses transactivation by FKHR (FOXO1) by multiple mechanisms. Direct and indirect effects on nuclear/cytoplasmic shuttling and DNA binding. Journal of Biological Chemistry 277 45276–45284. (10.1074/jbc.M208063200) 12228231

[bib56] Zhang T Cui H Yang Y Wu Z Gao H Yang F Xing M 2012 Effects of dietary protein levels on growth performance, digestibility of nutrients, and serum biochemical parameters in growing female minks. Chinese Journal of Animal Nutrition 5 835–844.

[bib57] Zhon M 2015 PhD thesis. Anhui, China: Anhui University of Chinese Medicine.

[bib58] Zhu J Liu W Yu J Zou S Wang J Yao W Gao X 2013 Characterization and hypoglycemic effect of a polysaccharide extracted from the fruit of Lycium barbarum L. Carbohydrate Polymers 98 8–16. (10.1016/j.carbpol.2013.04.057) 23987311

